# Food insecurity and social inequalities in households headed by older people in Brazil: a secondary cross-sectional analysis of a national survey

**DOI:** 10.1186/s12889-023-16332-0

**Published:** 2023-07-25

**Authors:** Eloah Costa de Sant’Anna Ribeiro, Camilla Christine de Souza Cherol, Rosana Salles da Costa, Paulo Cesar Pereira de Castro, Aline Alves Ferreira

**Affiliations:** Institute of Nutrition Josué de Castro, Federal University of Rio de Janeiro, Avenida Carlos Chagas Filho, 373, Bloco J, 2° andar, sala 18 – Cidade Universitária, Rio de Janeiro (RJ), CEP: 21941-902 Brazil

**Keywords:** Older adults, Food insecurity, Social inequalities, Brazil

## Abstract

**Background:**

The presence of food insecurity in households headed by older people is related to social inequalities. The objective of this study was to analyze the prevalence and factors associated with moderate/severe food insecurity in households headed by older people.

**Methods:**

A cross-sectional study based on a nationally representative sample of older adults aged ≥ 60 years was conducted using data from the 2017/2018 Family Budget Survey. In the study, moderate/severe food insecurity was the dependent variable, with food insecurity assessed with the Brazilian Household Food Insecurity Measurement Scale. Prevalence and odds ratio estimates were generated with 99% confidence intervals. Data analysis was performed using STATA software.

**Findings:**

A total of 16,314 households headed by older people were identified. Approximately 10.1% of these households were in the moderate/severe range for food insecurity. The majority are female (11.9%)and self-declared indigenous people (25.5%), with a lack of schooling (18.3%) and a per capita income of up to half of one minimum wage (29.6%). The analysis model found that color/race, region, schooling, per capita household income, and social benefits received in the household were statistically significant factors (p value < 0.01).

**Conclusion:**

Moderate/severe food insecurity in households headed by older people is associated with the pronounced social inequalities present in Brazil, and these findings intensify the need for additional study of the challenges faced by this age group.

**Supplementary Information:**

The online version contains supplementary material available at 10.1186/s12889-023-16332-0.

## Introduction

In 2015, the Sustainable Development Goals (SDGs),created by the United Nations (UN), called for the construction and implementation of public policies aimed at reducing inequities and improving population health worldwide by 2030. However, in recent years, several environmental, political, social and economic challenges have delayed the implementation of these goals, among which SDG 2 aimed to end hunger and guarantee access to safe, nutritious and sufficient food for all people, eradicating all forms of malnutrition [1, 2].

Instead of diminishing as hoped, hunger has increased worldwide [2]. According to the Food and Agriculture Organization (FAO) 2021 report, there have been numerous setbacks over the past six years regarding access to food in Latin America and the Caribbean, causing the prevalence of hunger to increase by 2% between 2019 and 2020. Brazil is among the Latin American countries with the highest increases in hunger [1].

In Brazil, public policies on Food and Nutrition Security (FNS) and the fight against hunger and poverty that were implemented in the 2000s have had an impact on the reduction of food insecurity (FI) levels, so much so that in 2014, the country was removed from the world hunger map. However, the population survey carried out in 2018 [3] pointed to a reversal of this achievement with a significant reduction in the proportion of food secure households and an increase in FI (from 22.6% to 2013 to 36.6% in 2018) [3, 4].

FI is characterized by the violation of regular and permanent access to quality food in sufficient quantities [5, 6], which affects the health and well-being of individuals. In older people, the impact of this burden is even greater [7]. In addition to the health conditions related to the aging process itself, FI can compromise the quality and quantity of food, especially for those living alone in poverty [8]. In addition, health conditions can limit mobility and increase social dependence in performing the activities of daily living (ADLs) [9].

From 2015 to 2020, in Latin America and the Caribbean, the growth rate of the older population (age ≥ 60 years), was 3.8%, which is higher than the rate expected worldwide [10]. According to data from the Brazilian Institute of Geography and Statistics (IBGE), from 2004 to 2015, the older population in Brazil increased from 9.8 to 14.3%. Projections indicate that in 2070, older people will represent 35% of the total population of the country [9, 10].

There has been an increase in life expectancy in Brazil in recent years as a result of advances in basic sanitary conditions, improved income, greater disease control and improved access to health care and technology [7]. However, the aging process is heterogeneous for many individuals, and the presence of numerous social inequalities for individuals 60 years or older hinders the effectiveness of public policies directed at this population. In addition, the aging process, when associated with the experience of hunger and social inequalities, can increase the dependence of older people on access to health services due to a greater chance of morbidity and mortality [9, 11].

Thus, the older population is a segment that needs social support, adequate food and policies that ensure good nutrition for active and healthy aging [7, 11]. Little is known about the profile of FI and hunger in this age group in the country. Therefore, this study aimed to analyze the prevalence and factors associated with moderate/severe FI in households headed by older people.

## Methods

### The survey

This cross-sectional, descriptive, analytical study was conducted with a sample of individuals ≥ 60 years of age based on a secondary analysis of public data from a nationally representative survey called the Family Budget Survey [Pesquisa de Orçamentos Familiares (POF)], which was conducted by the Brazilian Institute of Geography and Statistics [Instituto Brasileiro de Geografia e Estatística (IBGE)].

The POF was conducted from July 2017 to June 2018, with the objective of estimating the budget composition of households, analyzing the living conditions of the Brazilian population and examining related factors, such as regional disparities, family structure, nutritional profile and consumption of nutritious food [4].

Household data were obtained from the 2010 Demographic Census, which included the number of households in each census tract. The 2017–2018 POF estimates were weighted by the sample design and adjusted to compensate for the nonresponse of the investigated units. Data were collected in households through face-to-face interviews. Trained technical staff subjected the database to data quality control measures to assess information consistency [4]. The 2017–2018 POF used a complex sampling plan, by clusters, with a drawing of census tracts in the first stage, selected as primary sampling units (PSUs), and households in the second stage [4].

Altogether, 57,920 households were analyzed by trained interviewers, with face-to-face interviews conducted in the homes. The estimates were weighted considering the sample design, and necessary adjustments were made to compensate for any of the included units that did not respond [4].

### The older population

This study included individuals ≥ 60 years, whose age was estimated according to date of birth and the date of the interview; for respondents who did not know their date of birth, the reference age was used. In Brazil, individuals aged 60 years or older are classified as older people [12]. Thus, of the total number of individuals assessed in the POF, approximately 15% (n = 26,199) were considered older people, and 28.5% (n = 16,314) of the households surveyed were headed by older people. Only these households headed by older people were included in the analysis.

### Study variables

#### Household food insecurity

The Brazilian Household Food Insecurity Measurement Scale (EBIA) is the main tool for assessing household food insecurity (FI) in Brazil, which defines FI as limited access to safe and nutritious food of sufficient quality and quantity. It is a psychometric scale that assesses the interviewee’s perception of access to this food in the three months (90 days) prior to the interview. This scale was adapted and validated for the Brazilian population and has been used in national surveys since the Brazilian National Household Sample Surveys (PNAD) conducted in 2004 [13, 14].

The current version of the EBIA (2017–2018 POF) consists of 14 questions with dichotomous answers (yes, no) regarding access to food in the three months preceding its application. Eight items apply only to households with adults (aged 19 years or older), and 6 items apply exclusively to households with children and/or adolescents [13, 14]. The person within the family responsible for purchasing and preparing meals was the preferred interviewee in the 2017–2018 POF [4]. The classification of the EBIA establishes four mutually exclusive categories of food security and three levels of FI on the basis of recommended cutoffs for households with and without children and/or adolescents aged < 18: (i) food security (regular and permanent access to quality food in sufficient quantity); (ii) mild FI (concern or uncertainty about food availability); (iii) moderate FI (quantitative reduction of food intake and/or disruption in eating patterns); and (iv) severe FI (presence of food deprivation) [13, 14].

In this study, the outcome variable was the occurrence of the most severe forms of FI in households (moderate/severe FI), treated dichotomously (yes, no). More information has been added to the Supplementary Material.

#### Independent variables

The independent variables for the heads of households were age (60 to 80 years, and ≥ 80 years), sex (male, female), skin color/race self-classified (white, black, brown, yellow/Asian, indigenous), and education level in years (> 8 years of study, 1 to 8 years of study and no schooling). The variables selected to characterize households headed by older people were region (South, North, Northeast, Southeast, Midwest), area (urban, rural), number of residents in the household (1, 2 and ≥ 3 people in the household), number of older people in the household (1 and ≥ 2 seniors in the household), per capita monthly income categorized into minimum wage equivalencies (MW) (≤ 1/2 MW, 1/2 to 1 MW and > 1 MW) considering the value during the research period (based on the minimum wage in Brazil in 2018: US $297.20 or R$ 954 (Brazilian real), receipt of income transfer through social programs, such as Bolsa Família Program (yes, no) and Continuous Provision Benefit (yes, no), receipt of retirement/pension (yes, no).

### Data analysis

The mean and standard error (SE) were used for continuous variables, with Student’s t test performed to compare means. For categorical variables, the percentage and 99% confidence interval (99% CI) were determined, and the chi-square test (p < 0.01) was used to assess associations between variables and moderate/severe FI. All variables were tested for normality using the Kolmogorov‒Smirnov and Shapiro‒Wilk tests.

The logistic regression model was used to verify the odds ratio (OR) for the occurrence of moderate/severe FI (yes, no dichotomous outcome). All study variables were considered in the bivariate model and expressed as ORs and respective 99% CIs; however, those that had p values < 0.05 in the crude analysis were considered for the final model. In the adjusted model, variables with p values < 0.01 were considered significant. All analyses included age as a confounding variable. The analyses were performed using STATA software version 16.0 (StataCorp LP, College Station, United States) [15] with the Survey Data Analysis command (svy prefix) and considering the complex sample design of the study and the expansion factors provided by the IBGE.

### Ethical aspects

According to Resolution No. 466 of December 12, 2012, from the National Committee of Ethics in Research (CONEP), studies that use secondary data available in the public domain do not need approval by a local Ethics Committee CEP-CONEP System [16]. This study used data available in the public domain from the Brazilian Institute of Geography and Statistics. This research did not receive any specific grant funding from agencies in the public, commercial, or not-for- profit sectors.

## Results

It was observed that, on average, heads of households who were considered older people were 69.8 years old, had 6.4 years of schooling and a per capita household income of R$2720.59 (US $847.54), equivalent to approximately three per capita monthly incomes(data not presented in tables).

More than half of the heads of household were male (53.1%), self-declared white (50.1%) and most had 1 to 8 years of study (49.4%). The majority lived in the Southeast (46.9%) and approximately 85.6% lived in urban areas. A total of 38.2% of the households had ≥ 3 residents, and in most households, there was 1 older person (61.5%). Regarding total monthly family income,most households headed by older people received ≥ 1 minimum wage. The majority did not receive support from the Benefit of Continuous Provision (94%) or the Bolsa Família Program (95%). However, 82.2% received retirement and pensions, which are guaranteed by legislation for the older population (Table 1).

**Table 1:** Descriptive analysis of the households headed by older people. Family Budget Survey

Variables		Households headed by older people		
		n	_%_1	99% CI^2^
Age (years)	60 to 80	14,263	87.3	86.2; 88.3
	≥ 80	2,051	12.7	11.7;13.8
Sex	Male	8,872	53.1	51.7; 54.6
	Female	7,442	46.8	45.4; 48.3
Skin color/race	White	7,027	50.1	48.4; 51.7
	Black	1,792	10.4	9.6; 11.4
	Brown	7,272	38.1	36.6; 39.6
	Asian/Yellow	92	0.9	0.5; 1.4
	Indigenous	93	0.5	0.3; 0.7
Education level (years)	> 8	4,503	33.9	32.2; 35.6
	1 to 8	8,338	49.4	15.7; 17.8
	No schooling	3,473	16.8	47.7; 51.0
Region	South	2,468	15.4	14.6;16.3
	North	1,797	5.4	45.0; 5.9
	Northeast	5,514	25.7	24.6; 26.8
	Southeast	4,671	46.9	45.5; 48.3
	Midwest	1,864	6.5	5.94; 7.1
Area	Urban	12,413	85.6	84.6; 86.4
	Rural	3,901	14.4	13.5; 15.4
Number of residents in the household	1	3,774	23.9	22.6; 25.2
	2	5,981	37.9	36.5; 39.3
	≥ 3	6,559	38.2	36.8; 39.7
Number of older people in the household	1	10,152	61.5	60.0; 63.0
	≥ 2	6,162	38.5	37.0; 40.0
Per capita monthly income*	≥ 1 MW	12,036	78.3	77.0;79.5
	1/2 to 1 MW	3,225	16.8	15.7; 17.9
	≤ 1/2 MW	1,053	4.9	4.4; 5.5
Receipt of the Continuous Provision Benefit	No	15,160	94.0	93.4; 94.6
	Yes	1,154	6.0	5.4; 6.6
Receipt of the Bolsa Família Program	No	15,299	95.0	94.4; 95.6
	Yes	1,015	5.0	4.4; 5.5
Receipt of retirement/pension	Yes	13,298	82.2	81.1; 83.3
	No	3,016	17.7	16.7; 18.9

Notes: CI, confidence interval. MW, Minimum wage. ^1^Percentage; ^2^99% confidence interval.

*Minimum wage in Brazil in 2018: $US 297.20 (R$954 (Brazilian real)).

**Fig. 1:**
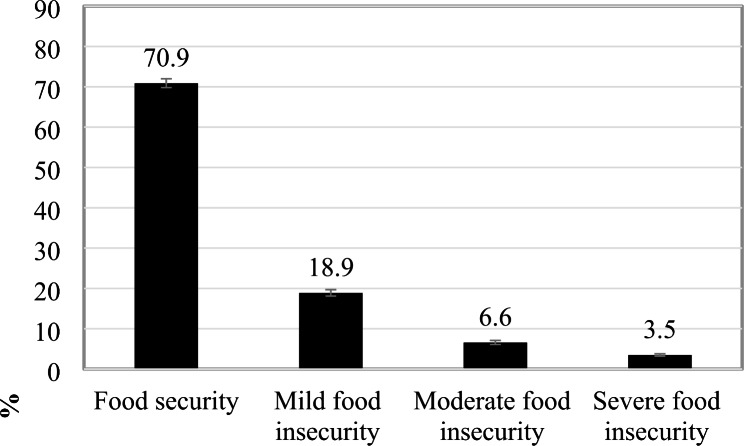
Prevalence of food insecurity in households headed by older people in Brazil. *Family Budget Survey*

FI was observed in 29% of households headed by older people, of which 10.1% (99% CI 9.29; 11.13) had severe forms of FI (moderate/severe) (Fig. 1).

Households headed by older people who declared themselves to be indigenous had a higher prevalence of moderate/severe FI (25.5%). Approximately 30.4% of households with FI were headed by black (16%) or brown (14.4%) older people; that is, they were considered black in Brazil. This finding was more than five times higher than the households facing FI headed by older white people (5.8%). The North had the highest prevalence of moderate/severe FI at 20.8%. Lower income strata and not receiving retirement/pensions were associated with higher proportions of moderate/severe FI (Table 2).

**Table 2:** Bivariate descriptive analysis of the association between sociodemographic variables and moderate/severe food insecurity in households headed by older people.*Family Budget Survey*

Variables		Moderate/severe food insecurity					p value^3^
		Yes			No		
		_%_1	99% CI^2^		_%_1	99% CI^2^	
Age (years)	60 to 80	10.8	9.8; 11.8		89.2	88.2; 90.2	0.000
	≥ 80	6.16	4.7; 8.0		93.8	92.0; 95.3	
Sex	Male	8.7	7.7; 9.8		91.3	90.2; 92.3	0.000
	Female	11.9	10.6; 13.3		88.1	86.7; 89.4	
Skin color/race	White	5.8	4.9; 6.8		94.2	93.2; 95.1	0.000
	Black	16.0	13.1; 19.4		84.0	80.6; 86.9	
	Brown	14.4	12.8; 16.0		85.6	84.0; 87.1	
	Asian/Yellow	2.8	0.8; 9.0		97.1	90.9; 99.1	
	Indigenous	25.5	13.4; 43.1		74.5	56.9; 86.6	
Educational level (years)	> 8	4.82	3.9; 6.0		95.2	94.0; 96.1	0.000
	1 to 8	11.1	9.8; 12.5		88.9	87.5; 90.2	
	No schooling	18.3	16.1; 20.8		81.6	79.2; 83.9	
Region	South	4.2	3.2; 5.4		95.8	94.6; 96.8	0.000
	North	20.8	17.1; 24.9		79.2	75.0; 82.9	
	Northeast	16.4	14.6; 18.4		83.6	81.6; 85.4	
	Southeast	7.0	5.8; 8.4		93.0	91.6; 94.2	
	Midwest	13.9	9.3; 20.3		86.1	79.7; 90.7	
Area	Urban	9.7	8.7; 10.7		90.3	89.3; 91.3	0.000
	Rural	13.2	11.1; 15.6		86.8	84.4; 88.9	
Number of residents in the household	1	10.7	8.9; 12.8		89.3	87.2; 91.1	0.000
	2	8.5	7.2; 10.1		91.4	89.9; 92.8	
	≥ 3	11.4	10.2; 12.9		88.5	87.1; 89.8	
Number of older people in the household	1	11.6	10.5; 12.7		88.4	87.2; 89.5	0.000
	≥ 2	8.0	6.8; 9.3		92.0	90.7; 93.2	
Per capita monthly income*	≥ 1 MW	7.0	6.1;7.9		93.0	92.1; 93.8	0.000
	1/2 to 1 MW	19.3	17.0; 21.9		80.7	78.1; 83.0	
	≤ 1/2 MW	29.6	25.1; 34.5		70.4	65.4; 74.9	
Receipt of the Continuous Provision Benefit	No	9.8	8.9; 10.7		90.2	89.3; 91.1	0.000
	Yes	16.7	13.3; 20.8		83.3	79.2; 86.7	
Receipt of the Bolsa Família Program	No	9.2	8.4; 10.2		90.8	89.8; 91.6	0.000
	Yes	28.3	23.4; 33.8		71.7	66.2; 76.6	
Receipt of retirement/pension	Yes	8.7	7.9; 9.7		91.3	90.3; 92.1	0.000
	No	16.9	14.6; 19.4		83.1	80.6; 85.4	

Notes: CI, confidence interval. MW, Minimum wage. ^1^Percentage; ^2^99% confidence interval. *Minimum wage in Brazil in 2018: $US 297.20 (R$954,Brazilian real); ^**3**^P-valor = Chi-square test

Considering the analyses of the final model (Table 3), households headed by older people who were Indigenous (OR = 3.5; 99% CI 1.3; 9.3), Black (OR = 1.8; 99% CI 1.3;2.4) and Brown (OR = 1;5; 99% CI 1.2; 1.9) were approximately twice as likely to be in moderate/severe IF situations. A low level of education had a higher OR for moderate/severe FI. Families residing in the Midwest (OR = 2.9; 99% CI 1.7; 5.0), North (OR = 2.8; 99% CI 2.0; 4.5) and Northeast (OR = 2.5; 99% CI 1.8; 3.5) were approximately three times more likely to be in a moderate/severe FI situation. With regard to income, those with a per capita income less than or equal to half of one MW were 2.6times more likely to be in a moderate/severe FI situation when compared to households with income greater than or equal to 1 MW (CI99% = 1.9;3.5). Furthermore, in households where the head did not receive income transfer programs/social benefits, especially retirements or pensions, the most severe levels of FI occurred (Table 3).

**Table 3:** Odds ratios (OR) and 99% confidence intervals (CI) of the association between sociodemographic variables and moderate/severe household food insecurityin households headed by older people. *Family Budget Survey*

Variables		Moderate/severe food insecurity						
		Bivariate model				Adjusted model		
		**OR**¹	99% CI **²**	**p value** ^**3**^		**OR**¹	99% CI **²**	**p value** ^**4**^
Sex	Male	1.0				1.0		
	Female	1.4	1.2; 1.7	0.000		1.5	1.2; 1.8	0.000
Skin color/race	White	1.0				1.0		
	Black	3.0	2.3; 4.1	0.000		1.8	1.3; 2.4	0.000
	Brown	2.7	2.2; 3.4	0.000		1.5	1.2; 1.9	0.000
	Asian/Yellow	0.5	0.1; 1.6	0.125		0.6	0.2; 2.4	0.248
	Indigenous	5.6	2.5; 12.6	0.000		3.5	1.3; 9.3	0.001
Educational level (years)	> 8	1.0				1.0		
	1 to 8	2.5	1.9; 3.3	0.000		2.0	1.5; 2.7	0.000
	No schooling	4.9	3.7; 6.4	0.000		2.7	2.0; 3.6	0.000
Region	South	1.0				1.0		
	North	6.0	4.1; 8.6	0.000		2.9	2.0; 4.5	0.000
	Northeast	4.6	3.4; 6.3	0.000		2.5	1.8; 3.5	0.000
	Southeast	1.7	1.2; 2.5	0.000		1.6	1.1; 2.2	0.002
	Midwest	3.7	2.1; 6.3	0.000		2.9	1.7; 5.0	0.000
Area	Urban	1.0						
	Rural	1.4	1.1; 1.8	0.000				
Number of residents in the household	1	1.0						
	2	0.8	0.6; 1.0	0.010				
	≥ 3	1.0	0.8; 1.3	0.732				
Number of older people in the household	1	1.0						
	≥ 2	1.5	1.2; 1.8	0.000				
Per capita monthly income*	≥ 1 MW	1.0				1.0		
	1/2 to 1 MW	3.1	2.6; 3.9	0.000		1.9	1.5; 2.3	0.000
	≤ 1/2 MW	5.6	4.3; 7.3	0.000		2.6	1.9; 3.5	0.000
Receipt of the Continuous Provision Benefit	No	1.0						
	Yes	1.9	1.4; 2.5	0.000				
Receipt of the Bolsa Família Program	No	1.0				1.0		
	Yes	3.8	2.9; 5.0	0.000		1.5	1.1; 2.1	0.000
Receipt of retirement/pension	Yes	1.0				1.0		
	No	2.0	1.7; 2.5	0.000		1.7	1.3; 2.1	0.000

Notes: CI, confidence interval. MW, Minimum wage. Age was included as a confounding variable. ^1^Odds Ratio; ^2^99% confidence interval.^3^p valor > 0.05; ^4^p valor > 0.01. *Minimum wage in Brazil in 2018: $US 297.20 (R$954, Brazilian real)

## Discussion

### Key findings

The results of the present study showed the magnitude of FI levels in households headed by older people. A lower prevalence of FI in the older population (29.1%) can be observed when compared to FI estimates for the Brazilian population in general, based on data from the same population survey [4]. However, the values were similar to the FI of households headed by older people in the 2004 PNAD (29.8%), the only national survey that has evaluated FI in the older population [17].

There are still few studies dedicated to studying the levels of estimated FI in households with older people. In the early 2010s, moderate/severe FI ranged from 17.1% among households headed by older people in the 2004 PNAD survey to 6.6% in Campinas, a municipality in the state of São Paulo [17, 18]. For other age groups, the development and implementation of programs and public policies aimed at combating extreme poverty and hunger are justified [18, 19].

In relation to other countries, in recent years, there has been an increase in the percentage of FI and world hunger. Brazil, among Latin American and Caribbean countries, had a lower prevalence of moderate/severe FI. However, this does not eliminate the presence of hunger in households headed by older people in the country [20–22]. The FI scenario in older adults in Brazil is far worse when compared to households in the United States and Australia. There is evidence that FI in households headed by older people in Brazil may be almost 20 times higher than that in Australia [23, 24].

In Brazil, the EBIA has helped to measure FI in the population since 2004, indicating increasing trends in all forms of FI from 2018 onward [25]. As in the present study, national surveys that evaluated FI in the older population also used the EBIA as an instrument of analysis, which makes possible the plural understanding of particular aspects of how this age group behaves within society. The presence of FI measured by the EBIA is considered a social indicator, and in the national territory, it was used in other population surveys, allowing reliable estimates of the prevalence of the violation of the right to food and nutrition security, as well as its association with socioeconomic and demographic aspects in older populations [17, 26, 27].

### Interpretation of results

The situation of moderate/severe FI in families with members and head of household ≥ 60 years of age is related to the heterogeneous distribution of income, access to education, sex and racial differences historically evidenced in Brazil. These findings tend to be consequences of the profound social inequalities and heterogeneity in aging that exists in Brazil [17, 28].

The socioeconomic and demographic relationship and FI in households headed by older people were similar to data from the adult population [17, 18]. However, it should be considered that the families of households headed by older people with moderate/severe FI, in addition to living with hunger, also face complications arising from the aging process itself, including prejudice, marginalization and social discrimination. Discriminatory attitudes toward older people directly affect their quality of life [28].

The factors associated with social inequalities and the FI situation in the older population are documented in the literature and have a generational effect on older individuals, accentuating the severity of the need to guarantee access to adequate food at home [3, 11, 26]. Among the evidenced inequalities, there was a higher prevalence of females (≥ 60 years of age) in a moderate/severe FI situation, indicating gender inequality. Authors have indicated greater availability of income in households headed by older men, one of the hypotheses being that these families have lower proportions of FI when compared to women [17, 29, 30]. The severe/moderate FI reveals itself in households headed by women as one of the consequences of sexism, even in socioeconomically favorable conditions, and is potentiated by the structural interaction of racism [31].

It is also noteworthy that there is a direct relationship between moderate/severe FI and ethnic-racial issues in Brazil. Historical, cultural, social and economic conditions have historically placed black and brown among the groups with the worst social and health indicators in the country. However, households with self-declared indigenous, black and brown older people had the highest prevalence of moderate/severe FI. Race/color in Brazil is a complex social construct and is highly related to health and food conditions, reflecting broader inequalities in the society [32, 33].

The level of education and FI have a consolidated relationship in the international literature, especially when analyzing the level of hunger,information, health and better job opportunities [3, 20, 34, 35]. Thus, it acts negatively on quality of life and healthy aging. Additionally, lower levels of education among older people may also indicate a greater need for them to continue working, even at older ages, to guarantee the necessary income for survival. Population studies have shown an association of inequality in access to health, especially among older people with less education who are residents of rural areas [17, 18, 36].

Regional inequalities are also associated with the FI situation, and the present findings reinforce hypotheses of the presence of high vulnerability in the northern part of the country [37, 38]. However, historically, the Southeast and South, which have low proportions of FI, are regions with more jobs, both in rural and urban areas, better living conditions and access to education, income, housing, health, food and income [38, 39].

In addition to evidence from other international and national surveys, this study found that the contribution of the older family members to household income was a protection mechanism [17, 33, 40]. It is understood that income has a direct relationship with the acquisition of food [18, 41]. Some studies point out that the financial stability of older people promotes security at home, placing the family in the context of food security [17, 18, 42]. In 2003, 75% of the family income was provided by individuals ≥ 60 years of age in households with one or more older persons, with most of the income of the older population coming from retirement and pensions [18]. This income can be considered a protective factor for the household due to the regularity of receipt of financial value; that is, a stable income is related to greater economic security, being inversely proportional to the presence of moderate/severe FI in families headed by older people [17, 40].

In this study, in households with severe forms of FI and whose income was complemented by receiving these benefits, the proportions were lower. Income transfer programs were considered important public policies for reducing poverty in Brazil. This result reinforces the importance of social policies aimed at guaranteeing the human right to adequate food as a way of guaranteeing access to food [43, 44]. The persistence of moderate/severe FI in the older population is associated with social disparities, and social support and the guarantee of programs and public policies aimed at meeting the needs of this population are fundamental [23, 25, 33].

### Strengths and limitations

The present study has some limitations, as the survival bias tends to reduce the associations found between the variables. Even so, because it has a robust design, it was possible to analyze a representative sample of households headed by older people and apply a validity instrument to measure FI, following a national trend. The present study was consolidated in an analysis in 2018, where in Brazil, a political and economic crisis was potentiated and aggravated during the COVID-19 pandemic [22, 45, 46]. It is necessary to carry out cohort studies in the future, offering the possibility of temporal analysis and investigating possible variations over time.

Being carried out at a population level, the study had an innovative impact on the analysis of risk factors associated with moderate/severe FI in households headed by older people in Brazil, which had not occurred since 2004 in Brazilian population studies. In addition, the magnitude of the consequences for public health was evidenced in public policies aimed at combating hunger, which is fundamental to understanding how to protect the future development of food and nutritional security and promote active and healthy aging in the country.

## Conclusion

To contribute to the knowledge of the FI situation of the Brazilian elderly population, especially those responsible for the households, the present study pointed out that the FI situation in households headed by older people was associated with women, black, brown and indigenous race/color, low levels of education, place of residence, and mainly lower income strata and nonreceipt of retirement/pension.

The literature on moderate/severe FI and aging is still limited, especially from the perspective of hunger. This may result from a perception of the association of income as the only indicator for hunger in individuals ≥ 60 years of age. However, there is a need for studies that integrate the areas of nutrition, public health and gerontology to fully capture the phenomena among this age group. Therefore, the definition of priorities and adjustments of measures to reduce FI, must be related to the magnitude of the effect of sociodemographic and regional factors.

It highlights the need to reformulate and elaborate policies and programs aimed at addressing FI in older adults to better meet their needs. In addition, interventions can help reduce FI. For example, conducting public policies aimed at creating programs that encourage older people to actively participate in food production, as well as social and health policies that promote preventive, restorative and development services for elderly individuals, that enable them to preserve their autonomy andremain active and useful member sofa society. In addition, it offers visibility to the older population group, with the aim of guaranteeing their basic human rights.

## Electronic supplementary material

Below is the link to the electronic supplementary material.

Supplementary Material 1

Supplementary Material 2

## Data Availability

Data sharing statement: This study analyzed public domain microdata from the Family Budget Survey (2018), made available by the Brazilian Institute of Geography of Statistics,https://www.ibge.gov.br/estatisticas/sociais/saude/24786-pesquisa-de-orcamentos-familiares-2.html?=&t=microdados.
