# Supplementary material for: Food insecurity and social inequalities in households headed by older people in Brazil: a secondary cross-sectional analysis of a national survey
Source: BMC Public Health. 2023 Jul 25;23:1424. doi: 10.1186/s12889-023-16332-0 (PMC10369831; doi:10.1186/s12889-023-16332-0)
Supplement: Supplementary file 1 — Supplementary Material 1 [file 12889_2023_16332_MOESM1_ESM.pdf]

Brazilian Household Food Insecurity Measurement Scale

Escala Brasileira de Insegurança Alimentar

Direct measurement scales of Food Insecurity (FI), such as the Brazilian Household Food Insecurity Measurement Scale, provide strategic information for the management of policies and social programs because they allow both identifying and quantifying the social groups at risk of FI as well as its determinants and consequences. It is a tool with an excellent cost-effectiveness ratio that has been used since the 1990s in several countries and whose application and analysis have shown to present common aspects to different sociocultural contexts and that represent the degrees of severity of FI: 1) psychological component – anxiety or doubt about the future availability of food in the house to meet the needs of the residents; 2) food quality – impairment of socially established preferences about food and its variety in the household stock; 3) quantitative reduction of food among adults; 4) quantitative reduction of food among children; 5) hunger – when someone goes the whole day without eating due to lack of money to buy food (COATES, 2006; PÉREZ-ESCAMILLA; SEGALL-CORRÊA, 2008; SWINDALE; BILINSKY, 2006). Based on the perception of the household's experience in the last 90 days, the Brazilian Household Food Insecurity Measurement Scale points to one of the following levels of FI experienced by households:

Table S1. Description of degrees of food security and insecurity.

Food security situation	Description
Food security	The family/household has regular and permanent access to quality food, in sufficient quantity, without compromising access to other essential needs.
Mild food insecurity	Concern or uncertainty about access to food in the future; inadequate food quality resulting from strategies that aim not to compromise food quantity.
Moderate food insecurity	Quantitative reduction in food intake among adults and/or disruption in eating patterns resulting from lack of food among adults.
Severe food insecurity	Quantitative reduction of food also among children, that is, disruption in eating patterns resulting from lack of food among all residents, including children. In this situation, hunger becomes an experience at home.

The Brazilian scale is an adapted and validated version of the one developed by the United States Department of Agriculture (USDA) in the mid-1990s. The Brazilian Household Food Insecurity Measurement Scale validation process was conducted by groups of researchers from universities located in the five regions of the country, coordinated by the Department of Collective Health of the State University of Campinas - UNICAMP, and required qualitative and quantitative studies to be carried out in urban and rural areas of the five major regions of the country, between 2003 and 2004.

In 2003, the first use of the EBIA was provided by the National Council for Scientific and Technological Development - CNPq, which financed a set of studies, through a project coordinated by UNICAMP, with the application of a scale to estimate the prevalence of AS in several Brazilian cities. In 2004, it was incorporated into the food security supplement of the National Household Sample Survey - PNAD. In 2006, this diagnosis was updated through the use of the EBIA in the National Survey of Demography and Health of Children and Women - PNDS, of the Ministry of Health.

The results of 2004 PNAD and 2006 PNDS confirm that FI is directly related to other socioeconomic factors and the composition of the household (such as, for example, the presence of residents under 18 years of age, the number of residents, gender or race of head of household, and household income). Therefore, it is advisable that the Brazilian Household Food Insecurity Measurement Scale questions are part of data collection instruments that also include socioeconomic, cultural and other variables that are deemed relevant for a more comprehensive characterization of the factors that affect the FS of families, as has been done by the Brazilian Institute of Geography and Statistics in specific PNAD supplements on the subject and, for the first time, as an integral part of the 2018 Family Budget Survey Assessment of Living Conditions questionnaire.

The EBIA analysis is based on a final score gradient resulting from the sum of affirmative responses to 14 questions. This score fits into the cutoff points (Table S2), which are equivalent to the theoretical constructs about food security, as shown in table S1.

Table S2. Cutoff points for households, with and without children under 18 years ofage, according to food security situation.

**Food security situation**	**Cutoff points for households**	
	With children under 18	No under 18 years old
Food security	0	0
Mild food insecurity	1-5	1-3
Moderate food insecurity	6-9	4-5
Severe food insecurity	10-14	6-8

These algorithms were updated during the technical workshop for the analysis of the EBIA, promoted at the time by the Secretariat for Evaluation and Information Management, of the then Ministry of Social Development and Fight against Hunger. Held between the 16th and 17th of August 2010, in Brasilia, the workshop had the participation of the research group that elaborated the EBIA. The technical group present at the workshop also approved some updates to the EBIA, which now has 14 questions in its composition. The component questions of the EBIA introduced in the 2018 Family Budget Survey can be seen in Table S3.

Table S3. Questions from the Brazilian Household Food Insecurity Measurement Scale.

Numbering	Question
**1**	In the past three months, have residents of this household been concerned that food would run out before they could buy or receive more food?
**2**	In the last three months, did the food run out before the residents of this household could afford to buy more food?
**3**	In the last three months, did the residents of this household run out of money to have a healthy and varied diet?
**4**	In the last three months, did the residents of this household eat only a few types of food they still had because they ran out of money?
**5**	In the last three months, did any household member aged 18 or over miss a meal because there was no money to buy food?
**6**	In the past three months, did any household member, age 18 or older, ever eat less than they thought they should because there was no money to buy food?
**7**	In the past three months, did any household member aged 18 or over ever feel hungry but not eat because there was no money to buy food?
**8**	In the past three months, did any household member aged 18 or over ever eat just one meal a day or go a whole day without eating because there was no money to buy food?
**9**	In the last three months, did any resident under 18 years of age ever fail to eat a healthy and varied diet because there was no money to buy food?
**10**	In the past three months, did any household member under the age of 18 ever eat less than they should because there was no money to buy food?
**11**	In the last three months, has the amount of food in the meals of any resident under 18 years of age been reduced because there was no money to buy food?
**12**	In the last three months, did any resident under 18 years of age ever skip a meal because there was no money to buy food?
**13**	In the past three months, did any household member under 18 years of age ever feel hungry but did not eat because there was no money to buy food?
**14**	In the last three months, did any resident under 18 years of age ever eat just one meal a day or go without food for a whole day because there was no money to buy food?

The structure of the scale with its questions constituting conceptual groupings and the described form of classification are conditions that allow estimating the prevalence of FS or FI in households in an adequate and scientifically tested manner, therefore, the use of analytical procedures to be used is not recommended. From one or more of your questions, alone, or any alternative. These approaches would provide results that are not consistent.

Bibliographic references:

Pesquisa de orçamentos familiares 2017-2018: análise da segurança alimentar no Brasil / IBGE, Coordenação de Trabalho e Rendimento. - Rio de Janeiro : IBGE, 2020. 65 p.

COATES, J. et al. Commonalities in the experience of household food insecurity across cultures: what are measures missing? The Journal of Nutrition, Bethesda: American Society for Nutrition - ASN, v. 136, n. 5, p. 1438S-1448S, May 2006. Suplemento. Disponível em: https://doi.org/10.1093/jn/136.5.1438S. Acesso em: ago. 2020.

PÉREZ-ESCAMILLA, R.; SEGALL-CORRÊA, A. M. Food insecurity measurement and indicators. Revista de Nutrição, Campinas: Pontifícia Universidade Católica - PUC-Campinas, v. 21, p. 15-26, jul./ ago. 2008. Suplemento. Disponível em: https://www.scielo.br/scielo. php?script=sci_arttext&pid=S1415-52732008000700003&lang=es. Acesso em: ago. 2020.

SWINDALE, A.; BILINSKY, P. Development of a universally applicable household food insecurity measurement tool: process, current status, and outstanding issues. The Journal of Nutrition, Bethesda: American Society for Nutrition - ASN, v. 136, n. 5, p. 1449-1452, May 2006. Suplemento. Disponível em: https://doi.org/10.1093/jn/136.5.1449S. Acesso em: ago. 2020.

Brasil, Ministério do Desenvolvimento Social e Combate à Fome. Nota Técnica DA/SAGI/MDS nº 128/2010: Relatório da Oficina Técnica para análise da Escala Brasileira de Medida Domiciliar de Insegurança Alimentar. Brasília: SAGI/DA, 30/08/2010.

SEGALL-CORRÊA, A. M. et al. Refinement of the brazilian household food insecurity measurement scale: recommendation for a 14-item EBIA. Revista de Nutrição, Campinas: Pontifícia Universidade Católica - PUC-Campinas, Mar./Apr. 2014.
