# Supplementary material for: Food insecurity and social inequalities in households headed by older people in Brazil: a secondary cross-sectional analysis of a national survey
Source: BMC Public Health. 2023 Jul 25;23:1424. doi: 10.1186/s12889-023-16332-0 (PMC10369831; doi:10.1186/s12889-023-16332-0)
Supplement: Supplementary file 2 — Supplementary Material 2 [file 12889_2023_16332_MOESM2_ESM.pdf]

AJE®

Editing Certificate

This document certifies that the manuscript

Food insecurity and social inequalities in households headed by older people in Brazil: a secondary cross-sectional analysis of a national survey.

prepared by the authors

Eloah Costa de Sant'Anna Ribeiro, Camilla Christine de Souza Cherol, Rosana Salles da Costa, Paulo Cesar Pereira de Castro Junior, Aline Alves Ferreira

was edited for proper English language, grammar, punctuation, spelling, and overall style by one or more of the highly qualified native English speaking editors at AJE.

This certificate was issued on **June 29, 2023** and may be verified on the AJE website using the verification code **1C93-2BE7-B7DB-DD37-3C6P**.

Neither the research content nor the authors' intentions were altered in any way during the editing process. Documents receiving this certification should be English-ready for publication; however, the author has the ability to accept or reject our suggestions and changes. To verify the final AJE edited version, please visit our verification page at aje.com/certificate. If you have any questions or concerns about this edited document, please contact AJE at support@aje.com.

AJE provides a range of editing, translation, and manuscript services for researchers and publishers around the world. For more information about our company, services, and partner discounts, please visit aje.com.
